# Duck Tembusu virus induces incomplete autophagy via the ERK/mTOR and AMPK/mTOR signalling pathways to promote viral replication in neuronal cells

**DOI:** 10.1186/s13567-023-01235-0

**Published:** 2023-11-07

**Authors:** Qing Wang, Yaqian Jiang, Guangbin Bao, Weiping Yao, Qing Yang, Shuyue Chen, Guijun Wang

**Affiliations:** Anhui Province Key Laboratory of Veterinary Pathobiology and Disease Control, College of Animal Science and Technology, Anhui Agricultural University, Hefei, 230036 China

**Keywords:** Duck Tembusu virus, incomplete autophagy, signalling pathways, viral replication, neuropathogenesis

## Abstract

Duck Tembusu virus (DTMUV) is a neurotropic virus in the genus *Flavivirus* that causes massive economic losses to the poultry industry in China and neighbouring countries. Autophagy is pivotal in cellular responses to pathogens and in viral pathogenesis. However, little is known about the roles of autophagy in DTMUV replication and viral pathogenesis, especially in neuropathogenesis. In this study, mouse neuroblastoma cells (Neuro-2a) were used to establish a cell model of DTMUV infection. Our experiments indicated that DTMUV infection induced incomplete autophagy in Neuro-2a cells. Then, we used different autophagy regulators to alter the autophagy induced by DTMUV and found that incomplete autophagy promoted DTMUV replication. Furthermore, we showed that DTMUV infection activated the ERK and AMPK pathways, resulting in decreased phosphorylation of the autophagy repressor mTOR, subsequently leading to autophagic induction. In addition, we utilized ICR mice in an animal model of DTMUV infection to evaluate the autophagic responses in brain tissues and investigate the effects of autophagy on viral replication and tissue lesions. Our results confirmed that DTMUV induced incomplete autophagy in mouse brain tissues and that autophagy inducer treatment promoted DTMUV replication and aggravated DTMUV-induced lesions, whereas autophagy inhibitor treatment had the opposite effects. In summary, DTMUV infection induced incomplete autophagy through the ERK/mTOR and AMPK/mTOR signalling pathways to promote viral replication in mouse neuronal cells, and DTMUV-induced incomplete autophagy contributed to the neuropathogenesis of DTMUV.

**Supplementary Information:**

The online version contains supplementary material available at 10.1186/s13567-023-01235-0.

## Introduction

Duck Tembusu virus (DTMUV) is a single-stranded RNA virus in the Flavivirus genus of the Flaviviridae family [1]. DTMUV infection causes severe encephalitis, decreased egg production and growth retardation in domestic ducks [2, 3]. An outbreak of DTMUV infection occurred in eastern China in April 2010 [4] and quickly spread to most duck farming areas in China, resulting in serious economic losses to the duck industry [5]. DTMUV has been reported to infect other species, such as geese, chickens, sparrows, pigeons and mice, and is even a potential threat to human beings, given its adaptation in wildlife and high homology to human pathogenic flaviviruses such as dengue virus (DENV), West Nile virus (WNV), Zika virus (ZIKV) and Japanese encephalitis virus (JEV) [6–8]. Similar to other flaviviruses, DTMUV has a genome of approximately 11 kb composed of an open reading frame encoding three structural proteins (capsid, membrane precursor, and envelope) and seven nonstructural proteins (NS1, NS2A, NS2B, NS3, NS4A, NS4B, and NS5) [1, 9]. Various types of avian cells (e.g., DF-1, HD11, DEF, and LSCC-BK3 cells) and a wide range of mammalian cells (e.g., Vero, BHK-21, A549, HeLa, and HEK293T cells) have been used to investigate the infection and pathogenic mechanisms of DTMUV [7]. BALB/c, Kunming and ICR mice can be used as animal infection models of DTMUV and show serious systemic and neurological symptoms [10–12].

Autophagy is a highly conserved cellular degradative process in eukaryotic cells that is associated with the removal of redundant or damaged organelles and proteins via the lysosomal degradative pathway to maintain homeostasis [13]. The processes of complete autophagy can be divided into three stages: the formation of autophagosomes and simultaneous capture of cytoplasmic cargoes, the fusion of autophagosomes with lysosomes to form autolysosomes, and the degradation of cargoes in autolysosomes [14]. Complete autophagy is termed autophagic flux. In incomplete autophagy, accumulated autophagosomes do not fuse with lysosomes for degradation, resulting in the blockage of autophagic flux [15]. Autophagy plays a “double-edged sword” role in virus‒host interactions [16]. Although host autophagy plays an antiviral role by degrading viruses directly or restricting the replication of viruses, some viruses have evolved strategies to interfere with autophagic processes to promote their own replication and propagation [17, 18]. The role of autophagy in flavivirus replication and pathogenesis is complex and has not been fully elucidated [14, 19]. For example, the capsid protein of WNV inhibits autophagy via AMPK degradation and contributes to the development of neurological disease [20], and JEV infection promotes autophagy while inhibiting autophagic flux, which is associated with microglial activation and cytokine production, resulting in neuroinflammation and brain dysfunction [21].

Recent studies have reported that DTMUV infection can induce complete autophagy in DEF, BHK21 and HEK293T cells, while autophagy facilitates viral replication [22–24]. To our knowledge, autophagic induction during virus infection has been found to be cell-type specific [19, 25]. DTMUV is neuroinvasive and neurovirulent [26]; however, whether DTMUV also induces complete autophagy in neurons and the roles of autophagy in DTMUV replication and neuropathogenesis are still not clear. In this study, a DTMUV infection cell model was constructed with mouse neuroblastoma cells (Neuro-2a). Western blotting, laser confocal microscopy and transmission electron microscopy (TEM) were used to determine the autophagy induced by DTMUV in Neuro-2a cells. Then, the role of autophagy in viral replication was examined through qRT-PCR and Western blotting. We also examined the potential signalling pathways of DTMUV-induced autophagy. In addition, mouse challenge experiments were performed to verify DTMUV-induced autophagy, and the roles of autophagy in DTMUV replication and neuropathogenesis were further evaluated.

## Materials and methods

### Viral strain and cell lines

The DTMUV strain AQ-19 (GenBank accession: MT708901.1) used in this study was isolated in our laboratory from a young goose with severe neurological dysfunction in Anhui Province, China, in 2019 [11]. Baby hamster kidney cells (BHK21, ATCC: CCL-10) and mouse neuroblastoma cells (Neuro-2a, ATCC: CCL-131) were cultured in Dulbecco’s modified Eagle’s medium (DMEM, Gibco, USA) containing 10% foetal bovine serum (FBS, Gibco, USA) at 37 °C in a humidified atmosphere of 5% CO_2_.

### Viral proliferation and infection

The DTMUV strain AQ-19 proliferated in BHK21 cells, and the viral particles were collected 72 h after infection. After repeated freezing and thawing at −80 °C, the samples were centrifuged at 12 000 × *g* for 15 min to remove the cell precipitate, and the supernatant virus solution was stored at −80 °C. The titre of AQ-19 was determined in BHK21 cells via the 50% tissue culture infective dose (TCID_50_) calculated by the Reed-Muench method. The titre of the viral stock was 10^–5.6^ TCID_50_/100 μL.

For DTMUV infection, Neuro-2a cells were grown to 70–80% confluence in 6-well plates, 24-well plates or 96-well plates and then infected with DTMUV AQ-19 at a multiplicity of infection (MOI) of 0.3 to establish a cell infection model based on our preliminary experiments and another study [23]. After 2 h of absorption, the cells were washed once with phosphate-buffered saline (PBS) and cultured in 2% FBS culture medium at 37 °C for 24 h, 36 h and 48 h. Indirect fluorescence assays (IFAs), Western blotting and absolute quantitative reverse transcription PCR (qRT-PCR) assays were performed to confirm viral infection and proliferation in Neuro-2a cells. Additionally, the viability of DTMUV-infected Neuro-2a cells was assessed.

### Cell viability assay

The Enhanced Cell Counting Kit-8 (CCK-8, Beyotime, China) was used to assess the viability of Neuro-2a cells as previously described [27] with some modifications. Briefly, Neuro-2a cells were cultured on 96-well plates at approximately 5 × 10^3^ cells per well for 24 h before infection with DTMUV AQ-19 for the defined incubation times. Cells were incubated with fresh culture medium with 10% (v/v) CCK-8 solution, and wells without cells were used as background. The 96-well plates were further incubated at 37 °C for 2 h in darkness. Subsequently, the absorbance at 450 nm was measured using an enzyme-linked immunosorbent assay reader (Thermo, USA). Assays were performed with four replicates and repeated as three independent experiments.

### RNA extraction and qRT-PCR

For analysis of the proliferation of DTMUV strain AQ-19, total RNA was extracted from goose or mouse brain tissues using a Total RNA Kit I (OMEGA, USA) and Neuro-2a cells using RNAiso Plus reagent (TaKaRa, Japan) according to the manufacturer’s instructions. Complementary DNA (cDNA) was synthesized using a BeyoRT TM cDNA First-Strand Synthesis Kit (Beyotime, China). Absolute qRT-PCR was conducted using ChamQ Universal SYBR qPCR Master Mix (Vazyme, China) on a 7500 Real-Time PCR System (Applied Biosystems, USA). The primers used in this study were forwards primer 5′-AGGAAGTGGAGCAATCAGGAA-3′ and reverse primer 5′-TAACAAGTGGCAGAGCAAAGG-3′. All qRT-PCR tests were performed in triplicate, and each set of qPCR assays was repeated three times.

### Immunofluorescence

Neuro-2a cells infected with AQ-19 for 24 h, 36 h and 48 h were washed with PBS, fixed with ice-cold methanol for 30 min at 4 °C, and then permeabilized with 0.2% Triton X-100 for 15 min. Cells were blocked with 5% skim milk for 1 h at 37 °C. Then, cell samples were incubated with DTMUV E protein monoclonal antibody (our laboratory preparation) for 1 h at 37 °C. After 4 washes with PBST, the cells were incubated with FITC-labelled goat anti-mouse IgG (Abbkine, USA) for 1 h at 37 °C in the dark. Next, the cells were stained for 5 min with DAPI (Solarbio, China) and observed under a fluorescence microscope (IX81, Olympus, Japan).

### Western blotting

Neuro-2a cells were washed twice with precooled PBS and lysed with radioimmunoprecipitation assay (RIPA, Solarbio, China) buffer with a protease phosphatase inhibitor mixture (Beyotime, China). Total protein was extracted from the brain tissues from goslings or mice with a Whole Cell Lysis Assay (KeyGEN Biotech, China) according to the instructions. The lysates were collected and centrifuged at 4 °C and 12 000 × *g* for 10 min for clarification, and the concentration of the extracted protein was determined using a BCA Protein Assay Kit (Thermo Fisher Scientific, USA). The equivalent protein samples were boiled in 5× SDS-PAGE loading buffer for 10 min, separated by SDS‒PAGE gels, and transferred to polyvinylidene fluoride (PVDF) membranes (Millipore, USA). Membranes were blocked for 2 h at 37 °C in Tris-buffered saline and Tween 20 (TBST) containing 5% nonfat milk. Then, the membranes were incubated with primary antibodies at 4 °C overnight. After TBST washes, the membranes were incubated with HRP-conjugated goat anti-mouse IgG or goat anti-rabbit IgG (Proteintech, China) at 37 °C for 1 h. The antibody-antigen complexes were visualized by an ECL Chemiluminescence Kit (Vazyme, China). The loading control in this study was β-actin. The grey values of the protein blots were measured by ImageJ software.

The primary antibodies used in this study were as follows: DTMUV E protein mouse monoclonal antibody (our laboratory preparation), LC3 rabbit polyclonal antibody (Proteintech, Cat No. 14600-1-AP; China), P62 rabbit polyclonal antibody (Proteintech, Cat No. 18420-1-AP; China), phospho-ERK rabbit mAb and ERK rabbit mAb (Cell Signaling Technology, Cat No. 4370 and Cat No. 4695; USA), phospho-AKT rabbit mAb and AKT rabbit mAb (Cell Signaling Technology, Cat No. 4060 and Cat No. 4691; USA), phospho-mTOR rabbit mAb and mTOR rabbit mAb (Cell Signaling Technology, Cat No. 5536 and Cat No. 2983; USA), phospho-AMPK rabbit mAb and AMPK rabbit mAb (Cell Signaling Technology, Cat No. 2535 and Cat No. 5831; USA) and β-actin mouse monoclonal antibody (Proteintech, Cat No. 66009-1-Ig; China).

### Transmission electron microscopy (TEM)

TEM is an important method widely used for monitoring autophagic induction [25]. Neuro-2a cells were seeded into 6-well plates and infected with the DTMUV strain AQ-19 for 36 h. Then, the cells were trypsinized and collected by centrifugation at 1000 × *g* for 10 min. In addition, brain samples from DTMUV-infected or uninfected mice were collected. The cell sediments and mouse brain samples were fixed with 2.5% glutaraldehyde at 4 °C overnight and sent to Wuhan Servicebio Company (Wuhan, China) for TEM observation. Autophagosome-like vesicles were observed under a JEM-1400 transmission electron microscope (JEM, Tokyo, Japan). Autophagosomes are identified as double-membrane vacuoles with diameters ranging from 0.2 to 1.0 μm [17].

### mRFP-GFP-LC3 transfection and autophagic flux measurements

For monitoring the progression from autophagosomes to autolysosomes, Neuro-2a cells were seeded onto glass coverslips and transfected with mRFP-GFP-LC3 plasmid using Lipofectamine 3000 (Invitrogen, USA) for 24 h according to the instructions and then infected with AQ-19 (0.3 MOI) for 36 h, and the changes in autophagic flux were visualized by laser confocal microscopy (Olympics, Japan). When autophagy occurs, the autophagosomes are labelled with both RFP and GFP, and bright yellow puncta (RFP+GFP+) can be seen. When autophagosomes fuse with lysosomes, resulting in GFP fluorescence quenching, only red puncta (RFP+) can be observed, indicating the formation of autolysosomes [28].

### Cell treatment

For analysis of the roles of autophagy in DTMUV replication, Neuro-2a cells were pretreated with rapamycin (RAPA; MCE, Cat No. HY-10219; USA) at 100 nM to trigger autophagy or 3-methyladenine (3-MA; MCE, Cat No. HY-19312; USA) at 1 mM to inhibit autophagy for 6 h before infection with DTMUV. For analysis of the signalling pathways that trigger autophagy, Neuro-2a cells were pretreated with the ERK inhibitor U0126 (20 μM; MCE, Cat No. HY-12031A; USA), AMPK inhibitor Compound C (10 μM; MCE, Cat No. HY-13418A; USA) or AKT activator SC79 (15 μM; MCE, Cat No. HY-18749; USA) for 2 h and then infected with DTMUV for the indicated times. The working concentration and incubation time of each drug were based on the cytotoxicities assessed using CCK-8 solution or determined by other references [23, 29].

### Animal experiments

Mice are widely used to study the pathogenesis of DTMUV [30], and we previously established an AQ-19 infection model using ICR mice [11]. Thirty-six 3-week-old female ICR mice were purchased from the Laboratory Animal Center of Anhui Medical University (China). Three days later, the mice were randomly divided into 4 groups (*n* = 9/group): the AQ-19, RAPA+AQ-19, 3-MA+AQ-19 and control groups. RAPA (2 mg/kg of body weight) and 3-MA (15 mg/kg of body weight) were administered 2 h prior to DTMUV AQ-19 challenge by intraperitoneal injection and then administered drugs every 24 h. The mice from the DTMUV infection and the control group were treated with saline. The mice from the AQ-19, RAPA+AQ-19 and 3-MA+AQ-19 groups were intracerebrally inoculated with 30 μL of AQ-19 viral stock, while the mice in the control group were intracerebrally inoculated with 30 μL of DMEM. The mice from the DTMUV-infected group were sacrificed at 2, 4, and 6 days post-infection (dpi). All mice were euthanized at 6 dpi, and the brain tissues were collected for Western blotting, qRT-PCR, TEM analysis, and immunohistochemical (IHC) or haematoxylin and eosin (H&E) staining. Pathological scores of brain lesions were recorded and analysed. All animal experiments were conducted using the protocol recommended by the Ethical Committee of Anhui Agricultural University (number AHAUB2022010).

To evaluate the autophagic responses induced by AQ-19 in the brain tissues of goslings, 2-day-old goslings were selected from a healthy herd from Anhui Province, and the goslings were not immunized. All goslings were confirmed to be negative for DTMUV, influenza virus, new castle virus, duck reovirus and goose parvovirus. Three days later, the goslings were randomly divided into 2 groups, which included the control group (4 goslings) and the AQ-19-infected group (8 goslings). According to our previous study [11], goslings were infected with AQ-19 through intramuscular (0.5 mL) inoculation or inoculated with DMEM through the same route. The goslings from the AQ-19-infected group were sacrificed at 3 and 5 days post-infection (dpi). All goslings were euthanized at 5 dpi, and the brain tissues were collected for Western blotting and qRT-PCR.

### Statistical analysis

Data are presented as the means ± standard deviations (SDs). The significance of the variability between different treatment groups was analysed by Student’s *t* test and one-way analysis of variance (ANOVA) with 95% confidence intervals using GraphPad Prism 8 software (La Jolla, CA, USA). A *P* value less than 0.05 was considered to be statistically significant.

## Results

### Infection of Neuro-2a cells with DTMUV

**Figure 1:**
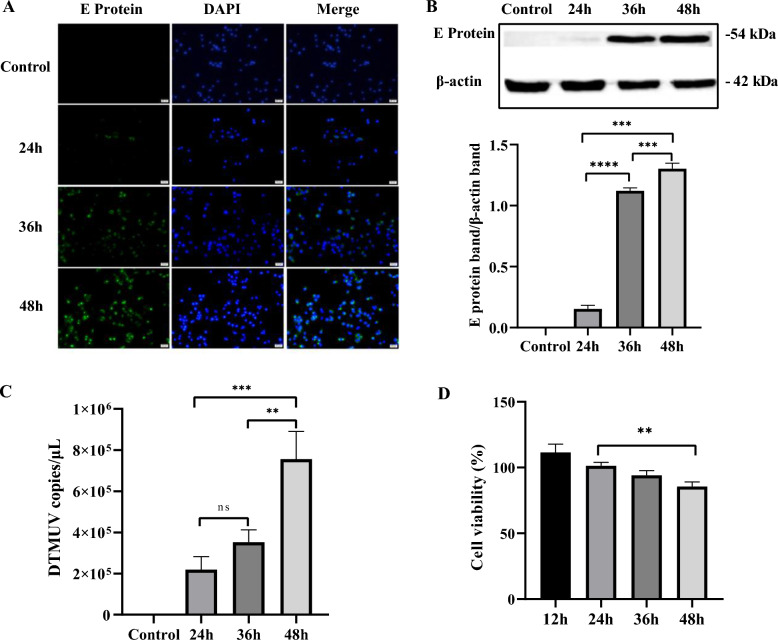
**DTMUV infection in Neuro-2a cells.** Neuro-2a cells were infected with DTMUV for 24 h, 36 h and 48 h. **A** Detection of DTMUV infection in Neuro-2a cells by indirect immunofluorescence assays. Green fluorescence indicates that cells were infected with DTMUV strain AQ-19. Scale bar, 20 μm. **B** Western blotting analysis of DTMUV infection. E protein levels were detected, and band intensities were analysed using ImageJ. **C** qRT-PCR detection of DTMUV proliferation. Total RNA of cells was extracted for measurement of viral copies through qRT-PCR assay. **D** The viability of Neuro-2a cells was evaluated by CCK-8 assays. The results are shown as the means ± SDs of three independent experiments. Significant differences were determined with one-way ANOVA. ns: not significant; **P < 0.01; ***P < 0.001; ****P < 0.0001.

For the establishment of an in vitro DTMUV-infected cell model, the susceptibility of Neuro-2a cells to AQ-19 infection was evaluated. AQ-19 infection in Neuro-2a cells was examined by IFA. Specific fluorescence was distributed in the cells of the infected group, and the fluorescence level increased with increasing infection time, while no signal for the control appeared, indicating that DTMUV had the potential to infect Neuro-2a cells (Figure 1A). To further confirm the replication of DTMUV AQ-19 in cells, we collected cell proteins for Western blotting, and total RNA was extracted for qRT-PCR analysis. Western blotting results showed that the expression of E protein in the infection group gradually increased with increasing infection time, indicating that AQ-19 can replicate in Neuro-2a cells (Figure 1B). Moreover, the qRT-PCR results confirmed that viral copy numbers increased from 24 to 48 h (Figure 1C). These results demonstrated that DTMUV AQ-19 can infect and replicate in Neuro-2a cells. Additionally, cell viability was evaluated, as shown in Figure 1D. The viability of DTMUV-infected Neuro-2a cells decreased gradually from 24 to 48 h post-infection (hpi).

### DTMUV infection induces incomplete autophagy in Neuro-2a cells

**Figure 2:**
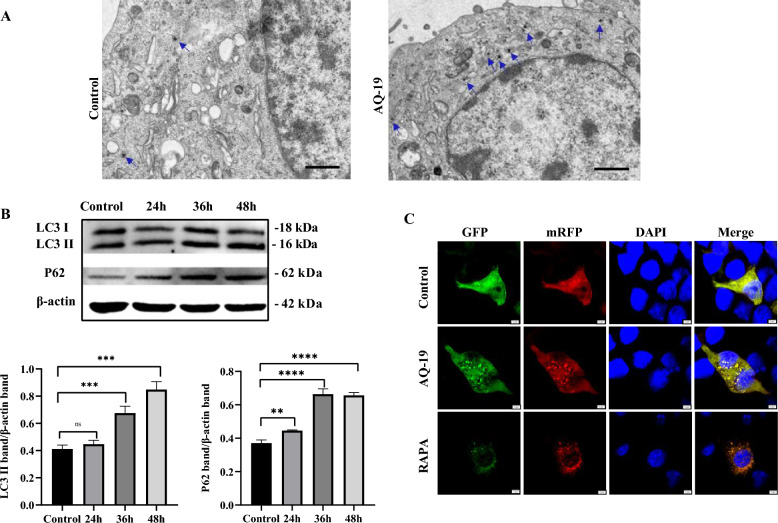
**Incomplete autophagy is induced by DTMUV infection in Neuro-2a cells. A** Uninfected Neuro-2a cells and Neuro-2a cells infected with DTMUV for 36 h were observed by TEM. Autophagosome-like vesicles are indicated with blue arrows. Scale bar, 1 μm. **B** Uninfected Neuro-2a cells and Neuro-2a cells infected with DTMUV for 24 h, 36 h and 48 h. The expression of the autophagy-related proteins LC3-II and P62 was determined by Western blotting, and band intensities were analysed. The results represent the means ± SDs of three independent experiments. Significant differences were determined with one-way ANOVA. ns: not significant; **P < 0.01; ***P < 0.001; ****P < 0.0001. **C** Neuro-2a cells transfected with the mRFP-GFP-LC3 plasmid were uninfected or infected with DTMUV for 36 h, and fluorescent LC3 puncta were observed under a fluorescence microscope. Scale bar, 5 μm.

TEM is widely used for monitoring autophagy [31]. More autophagosome-like vesicles were observed in the DTMUV AQ-19-infected cells than in the uninfected cells (Figure 2A), indicating that AQ-19 infection can induce autophagy in Neuro-2a cells. In addition, Neuro-2a cells were infected with AQ-19 (0.3 MOI) for 24 h, 36 h and 48 h, and the levels of the autophagic marker protein LC3 and the cargo receptor for autophagic degradation P62 were determined by Western blotting. As shown in Figure 2B, AQ-19 infection increased the formation of LC3-II, and P62 accumulated, especially at 36 hpi and 48 hpi. These results suggested that DTMUV infection may induce incomplete autophagy in Neuro-2a cells.

Afterwards, Neuro-2a cells were transfected with a tandem-tagged fluorescent reporter to further explore the autophagic flux induced by AQ-19 infection. The results showed that AQ-19 infection induced colocalization of RFP and GFP signals, resulting in yellow puncta. However, with rapamycin treatment, the cells underwent complete autophagic flux and mainly showed red fluorescence (Figure 2C). These results further implied that AQ-19 infection induced incomplete autophagy in Neuro-2a cells.

### Incomplete autophagy promotes DTMUV replication

**Figure 3:**
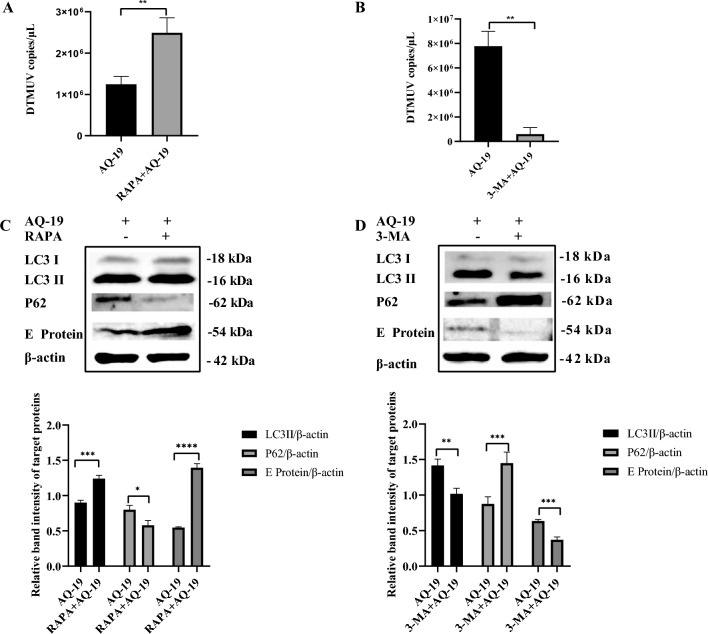
**Incomplete autophagy promotes the replication of DTMUV in Neuro-2a cells.** Neuro-2a cells were pretreated with RAPA or 3-MA to induce or inhibit autophagy before being infected with DTMUV for 36 h. **A**, **B** Total RNA of cells was extracted, and DTMUV copies were evaluated by qRT-PCR. **C**, **D** Cell lysates were harvested, and the levels of DTMUV E protein were evaluated by Western blotting. Band intensities were calculated and analysed using ImageJ software. The results are the means ± SDs of three independent experiments. Differences were evaluated using Student’s *t* test. *P < 0.05; **P < 0.01; ***P < 0.001; ****P < 0.0001.

To further investigate the role of autophagy in DTMUV replication, we pretreated Neuro-2a cells with RAPA or 3-MA to induce or inhibit autophagy and then infected the Neuro-2a cells with DTMUV at an MOI of 0.3 for 36 h. RAPA is an autophagy inducer that can promote autophagy by inhibiting mTOR; 3-MA is an inhibitor of class III phosphatidylinositol 3-kinase and was used to inhibit the formation of autophagosomes. The total RNA of the cells was extracted, and the expression levels of the viral NS5 gene were measured by qRT-PCR. As shown in Figure 3A, B, the levels of the NS5 gene were significantly higher in the RAPA-pretreated group than in the untreated group, and the levels of the NS5 gene were significantly lower in the 3-MA-pretreated group than in the untreated group. In addition, total protein samples at 36 hpi were collected, and the expression of E protein, LC3 and P62 was detected by Western blotting. As shown in Figure 3C, RAPA treatment increased the expression of LC3-II and decreased the expression of P62; the E protein level in the RAPA-pretreated group was significantly higher than that in the untreated group. Consistently, treatment with 3-MA decreased the expression of LC3-II and increased the expression of P62; the E protein level in the 3-MA-pretreated group was significantly lower than that in the untreated group (Figure 3D). The above results indicated that DTMUV-induced autophagy promotes viral replication.

### Activation of the ERK-mTOR and AMPK-mTOR signalling pathways was involved in DTMUV-induced autophagy

**Figure 4:**
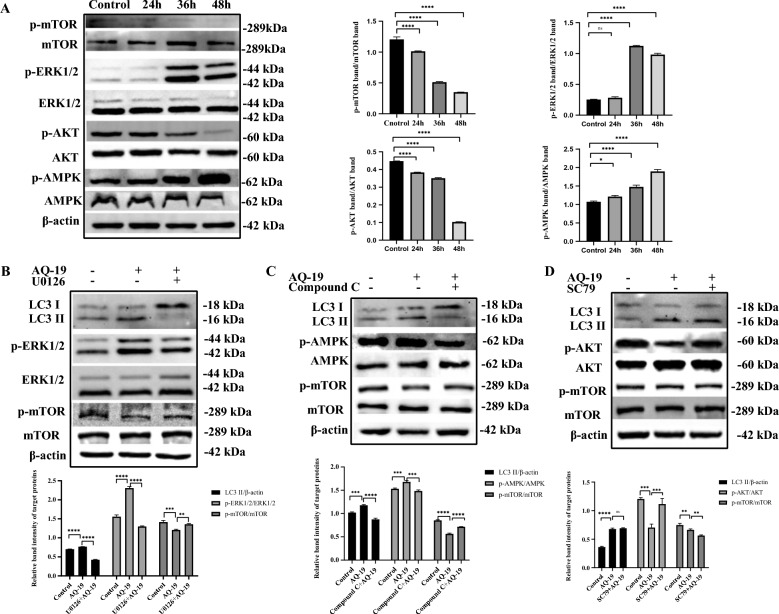
**DTMUV induces autophagy in Neuro-2a cells by activating the ERK/mTOR and AMPK/mTOR signalling pathways. A** Uninfected Neuro-2a cells and Neuro-2a cells infected with DTMUV for 24 h, 36 h and 48 h. The phosphorylation levels of ERK1/2, AMPK, AKT and mTOR were determined by Western blotting. **B**–**D** Uninfected Neuro-2a cells and Neuro-2a cells infected with DTMUV for 36 h after treatment with or without the ERK inhibitor U0126, AMPK inhibitor Compound C or AKT activator SC79. The expression levels of p-ERK, p-AMPK, p-AKT, LC3-II, and p-mTOR were evaluated by Western blotting. Band intensities were calculated and analysed using ImageJ software. The results are presented as the means ± SDs of three independent experiments. Significant differences were determined with one-way ANOVA. ns: not significant; *P < 0.05; **P < 0.01; ***P < 0.001; ****P < 0.0001.

mTOR is the main hub of the autophagy regulatory network, and the inactivation of mTOR promotes autophagy [32]. AMPK, ERK and AKT are upstream regulators of mTOR, which can regulate autophagy by regulating the phosphorylation of mTOR [29, 33, 34]. We detected the phosphorylation levels of ERK1/2, AKT, AMPK and mTOR in DTMUV-infected Neuro-2a cells at different time points. The phosphorylation of ERK1/2 and AMPK was significantly promoted, and the phosphorylation of AKT and mTOR was inhibited (Figure 4A).

To further explore whether the phosphorylation level changes of ERK1/2, AMPK and AKT were related to autophagy, we pretreated Neuro-2a cells with U0126 (inhibitor of ERK1/2), Compound C (inhibitor of AMPK) or SC79 (activator of AKT) before infection with DTMUV AQ-19, and the expression of LC3-II and phosphorylation of ERK1/2, AMPK, AKT and mTOR were evaluated. The results showed that the phosphorylation of ERK1/2 and AMPK decreased after treatment with the corresponding inhibitors, and the phosphorylation of AKT increased after treatment with the corresponding activator (Figures 4B–D). The expression of LC3-II was significantly decreased, and mTOR phosphorylation was re-established after pretreatment of DTMUV-infected cells with U0126 and Compound C (Figures 4B, C). However, the activation of AKT did not affect the level of LC3-II, indicating that AKT had no effect on DTMUV-induced autophagy in Neuro-2a cells (Figure 4D). These results suggested that DTMUV induces autophagy through the ERK/mTOR and AMPK/mTOR signalling pathways in Neuro-2a cells.

### DTMUV-induced incomplete autophagy promotes viral replication in mouse brain tissues and contributes to viral neuropathogenesis

**Figure 5:**
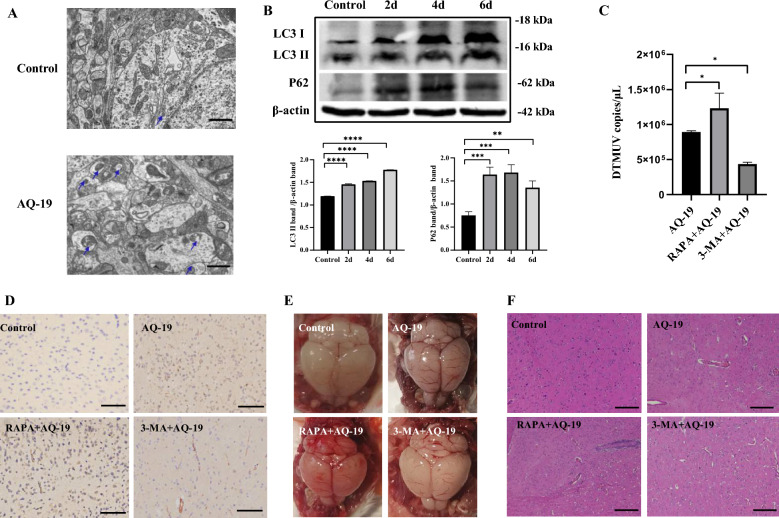
**DTMUV infection induces incomplete autophagy in the mouse brain, which contributes to viral replication and neuropathogenesis. A** Autophagosome-like vesicles (blue arrows) were observed in the mouse brains by TEM. Scale bar, 1 μm. **B** Protein levels of LC3-II and P62 in the brain tissues of mice infected with DTMUV for 2 dpi, 4 dpi and 6 dpi were determined by Western blotting, and band intensities were analysed. **C** qRT-PCR analysis of DTMUV copies in the brains of mice from the AQ-19, RAPA+AQ-19 and 3-MA+AQ-19 groups. **D** Immunohistochemical (IHC) staining was used to observe the distribution of DTMUV antigen (brown) in the brains of mice from the control, AQ-19, RAPA+AQ-19 and 3-MA+AQ-19 groups. Scale bar, 100 μm. **E** Histopathological examination of the brain tissues of mice from the control, AQ-19, RAPA+AQ-19 and 3-MA+AQ-19 groups. Scale bar, 200 μm. The results are presented as the means ± SDs of three experiments. Differences were analysed using one-way ANOVA. *P < 0.05; **P < 0.01; ***P < 0.001; ****P < 0.0001.

Having demonstrated the induction of incomplete autophagy in DTMUV-infected Neuro-2a cells, we further confirmed the results in an ICR mouse infection model. TEM and Western blotting were used to evaluate the autophagy induced by AQ-19 infection. The TEM results showed that the number of autophagosomes in brain tissues significantly increased after DTMUV infection (Figure 5A). Compared with that of the control group, the LC3-II expression in brain tissues from the AQ-19-infected mice was increased at 2 dpi and was sustained to 6 dpi, and the P62 protein accumulated throughout the infection experiment (Figure 5B). These results demonstrated that DTMUV strain AQ-19 triggers incomplete autophagy in the brain tissues of mice, which was consistent with the in vitro results.

qRT-PCR and IHC assays were performed to evaluate the role of autophagy in DTMUV replication in mouse brain tissues. As shown in Figure 5C, the viral copies in mouse brain tissues from the RAPA+AQ-19 group were significantly higher than those in the AQ-19 infection group, and the viral copies in brain tissues from the 3-MA+AQ-19 group were significantly lower than those in the DTMUV infection group. Consistently, IHC staining showed that the signals in mouse brain tissues from the RAPA+AQ-19 group were stronger than those in the 3-MA treatment group and untreated group, and the signals in the 3-MA+AQ-19 group were the weakest (Figure 5D), indicating that 3-MA treatment inhibited the replication of DTMUV in mouse brain tissues. H&E staining showed lymphoid perivascular cuffing and perivascular lymphoid infiltration in the brain tissues of mice from the RAPA+AQ-19 group, and the lesions were more severe than those in the DTMUV infection group and the 3-MA+AQ-19 group; moreover, the brain lesions in the 3-MA+AQ-19 group were less severe than those in the AQ-19 group (Figures 5E, F and Additional file 1). These results indicated that DTMUV-induced autophagy promotes viral replication in mouse brain tissues and enhances viral neuropathogenesis.

Additionally, DTMUV-induced incomplete autophagy was confirmed in AQ-19-infected goose brain tissues. Five-day-old goslings were challenged with AQ-19 through intramuscular injection. Depression was observed at 3 dpi, and paralysis and opisthotonos were observed at 5 dpi (data not shown). The brain tissues were collected at 3 dpi and 5 dpi. Western blotting was used to evaluate the autophagy induced by AQ-19 infection. qRT-PCR assays were performed to detect viral copies. As shown in Additional file 2A, LC3-II expression in brain tissues from AQ-19-infected goslings was increased at 5 dpi, and the P62 protein accumulated. As shown in Additional file 2B, viral copies in goose brain tissues could be detected at 3 dpi and were significantly increased at 5 dpi. These results indicated that DTMUV strain AQ-19 triggers incomplete autophagy in the brain tissues of goslings.

## Discussion

Autophagy is an essential and conserved intracellular degradative process that maintains cellular homeostasis by removing damaged organelles, aggregated proteins, excess lipids, and infectious microorganisms [13]. Flaviviruses are a genus of viruses that endanger human health worldwide [14]. Although the interactions between flavivirus infection and autophagy have been investigated in a number of studies, the overall role of autophagy in flavivirus replication and pathogenicity is complex and has not been elucidated [14, 35]. Previous studies have shown that DTMUV infection can induce autophagy and promote virus replication [23, 24]. In this study, we verified that DTMUV-induced autophagy promoted viral replication, and we found that DTMUV caused incomplete autophagy in neurocytes, which enhanced viral neuropathogenicity.

Neuro-2a cells are widely used to explore the pathogenic mechanism of neurotropic viruses, such as JEV, Pseudorabies virus and porcine haemagglutinating encephalomyelitis virus [21, 36, 37]. In this study, Neuro-2a cells were used to establish a DTMUV infection cell model, and we found that the DTMUV strain AQ-19 could infect and proliferate in Neuro-2a cells. Autophagic flux measurement results showed that the accumulation of autophagosomes occurred in AQ-19-infected Neuro-2a cells, and the degradation of P62 was prevented, which indicated that autophagic integrity was impaired. Our results were different from the results of DTMUV infection in DEF, BHK21 and HEK293T cells, in which DTMUV induced complete autophagic flux [22–24]. The reason may be due to cell-type differences. Peng et al. reported that different autophagic flux responses were oriented from different cells infected with the same strain of rabies virus [25]. Additionally, viral pathogens can manipulate autophagic processes to benefit their life cycles [16–18]. In the current study, the autophagy activator RAPA promoted DTMUV replication, and the autophagy inhibitor 3-MA inhibited the replication of DTMUV, which is consistent with previous studies [23, 24]. However, which DTMUV protein induced autophagy in Neuro-2a cells in our study requires further study.

Pathogens can regulate autophagy by a number of different signalling pathways. For instance, *Mycoplasma hyopneumoniae* induced incomplete autophagy in porcine alveolar macrophages through the JNK and AKT signalling pathways [38]. Respiratory syncytial virus induces autophagy to promote viral replication through the AMPK/mTOR signalling pathway in HEp-2 cells [17]. Some studies have reported the signalling pathways of autophagy induced by other flaviviruses. For instance, ZIKV infection of human foetal neural stem cells can trigger autophagy by inhibiting the AKT/mTOR signalling pathway [39]. ZIKV infection of human umbilical vein endothelial cells can modulate the AKT/mTOR and BECN1 signalling pathways to induce autophagy and enhance viral replication [40]. DENV was shown to induce autophagy by the BECN1/BCL2 pathway in A549 cells [41]. In this study, we examined the signalling pathways of autophagy induced by DTMUV, and the results indicated that DTMUV could induce autophagy in Neuro-2a cells via the ERK/mTOR and AMPK/mTOR signalling pathways. However, Zhao et al. reported that DTMUV induced autophagy via the ERK and PI3K-AKT-mTOR signalling pathways in duck embryo fibroblasts [23], which is different from our results. The AKT/mTOR signalling pathway is known to negatively regulate autophagy [39]. In this study, the phosphorylation level of AKT decreased after AQ-19 infection; however, treatment with the AKT activator SC79 did not inhibit autophagy, indicating that the inactivation of AKT is not essential for autophagy induction caused by AQ-19. In addition to regulating autophagy, downregulation of the AKT signalling pathway is associated with reduced cell viability and proliferation [42, 43], and AQ-19 infection-induced inactivation of AKT might be the reason for the decreased viability of Neuro-2a cells.

DTMUV, a neurotropic virus, acts on the central nervous system and causes neurological symptoms [44]. More autophagosomes were observed in the DTMUV-infected mouse brain, and P62 protein accumulated during the experiment, indicating DTMUV-induced incomplete autophagy, which further verified the results in vitro. To determine the effect of autophagy on the severity of DTMUV infection, mice were injected intraperitoneally with the autophagy inducer RAPA and inhibitor 3-MA. The viral load in the mouse brain tissues in the 3-MA+AQ-19 group was lower than that in the AQ-19 and RAPA+AQ-19 groups, and the histopathological changes in brain tissues in the 3-MA+AQ-19 group were slight compared with those in the AQ-19 and RAPA+AQ-19 groups. Similarly, autophagy inhibitors could reduce JEV infection and weaken the neurological signs and pathological changes in mouse brains, indicating the positive role of autophagy in the pathogenesis of JEV infection [45]. During JEV infection, incomplete autophagy promotes proinflammatory cytokine production and might contribute to the death of neuronal cells, which further causes neuronal dysfunction [21]. However, in WNV infection, the inhibition of autophagy through AMPK degradation induces the accumulation of protein aggregates in the brains of mice, contributing to the development of neurological disease [20]. Our study provides the first evidence that autophagy is associated with the neuropathogenicity of DTMUV infection.

In conclusion, this study demonstrated for the first time that DTMUV induced incomplete autophagy in Neuro-2a cells and mouse brain tissues, promoting the replication of DTMUV and contributing to the neuropathogenicity of DTMUV. It was also found that this incomplete autophagy occurs via activation of the ERK/mTOR and AMPK/mTOR pathways. This study broadens our understanding of the interaction between DTMUV infection and autophagy and provides new insights into the pathogenesis of DTMUV. However, further study is needed to explore the roles of autophagy in DTMUV infection in ducks, which must be understood to control DTMUV infections.

### Supplementary Information

**Additional file 1: Histological score of brain tissues in mice.** The histological score of mouse brain tissues was determined to be normal = 0, mild = 1, moderate = 2, severe = 3 or very severe = 4 based on the pathological changes. Data are represented as the mean ± SD of three biological replicates. Significant differences were calculated using one-way ANOVA. *P < 0.05; ***P < 0.001.

**Additional file 2: DTMUV AQ-19 infection induces incomplete autophagy in the goose brain.** **A** Protein levels of LC3-II and P62 in the brain tissues of geese infected with DTMUV for 3 dpi and 5 dpi were determined by Western blotting, and band intensities were analysed. **B** qRT-PCR analysis of DTMUV copies in the brains of geese infected with AQ-19. The results are presented as the means ± SDs of three experiments. Differences were analysed using one-way ANOVA (A) and Student’s *t* test (B). ns: not significant; ***P < 0.001; ****P < 0.0001.

## Data Availability

The data that generated during the current study are available from the corresponding author on reasonable request.

## Abbreviations

DTMUV: Duck Tembusu virus
DENV: Dengue virus
WNV: West Nile virus
ZIKV: Zika virus
JEV: Japanese encephalitis virus
Neuro-2a: mouse neuroblastoma cells
TCID_50_: 50% Tissue culture infective dose
MOI: multiplicity of infection
FBS: foetal bovine serum
PBS: phosphate-buffered saline
IFA: indirect fluorescence assay
qRT-PCR: quantitative reverse transcription PCR
PVDF: polyvinylidene fluoride
TBST: Tris-buffered saline and Tween 20
TEM: transmission electron microscopy
RAPA: rapamycin
3-MA: 3-methyladenine
dpi: days post-infection
hpi: hours post-infection
H&E: haematoxylin and eosin
SDs: standard deviations
ANOVA: one-way analysis of variance
