# Supplementary figures and images for: Duck Tembusu virus induces incomplete autophagy via the ERK/mTOR and AMPK/mTOR signalling pathways to promote viral replication in neuronal cells
Source: Vet Res. 2023 Nov 7;54:103. doi: 10.1186/s13567-023-01235-0 (PMC10631066; doi:10.1186/s13567-023-01235-0)

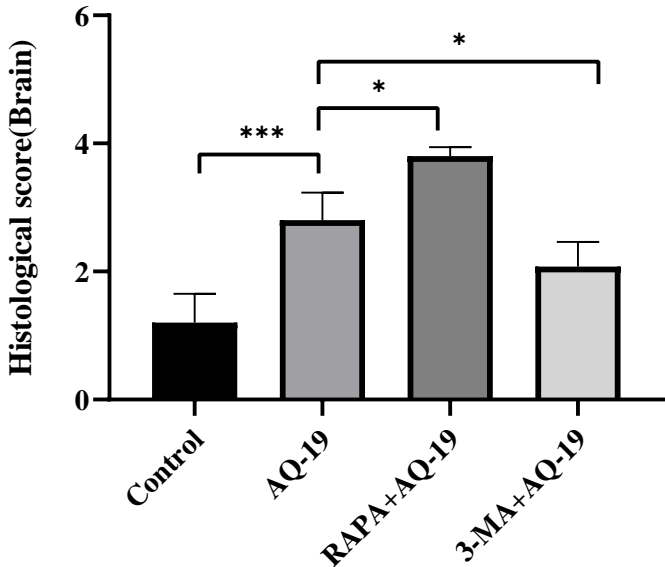

Supplement: Supplementary file 1 — Additional file 1: Histological score of brain tissues in mice. The histological score of mouse brain tissues was determined to be normal = 0, mild = 1, moderate = 2, severe = 3 or very severe = 4 based on the pathological changes. Data are represented as the mean ± SD of three biological replicates. Significant differences were calculated using one-way ANOVA. *P < 0.05; ***P < 0.001. [file 13567_2023_1235_MOESM1_ESM.pdf]

A

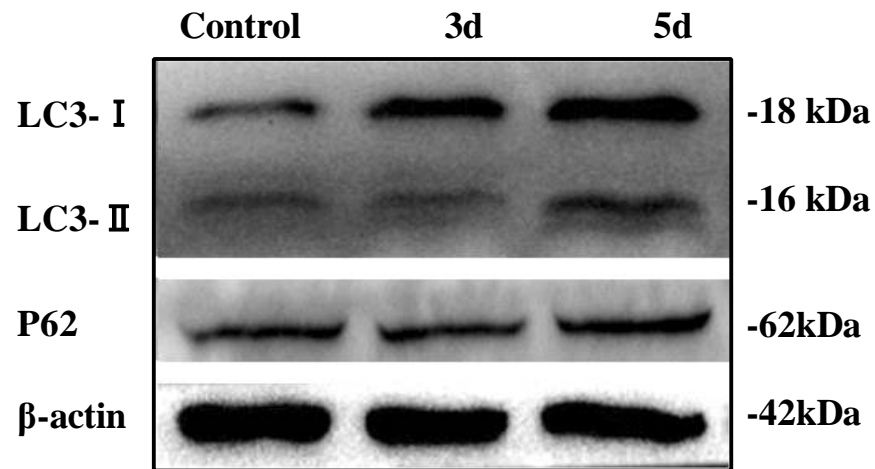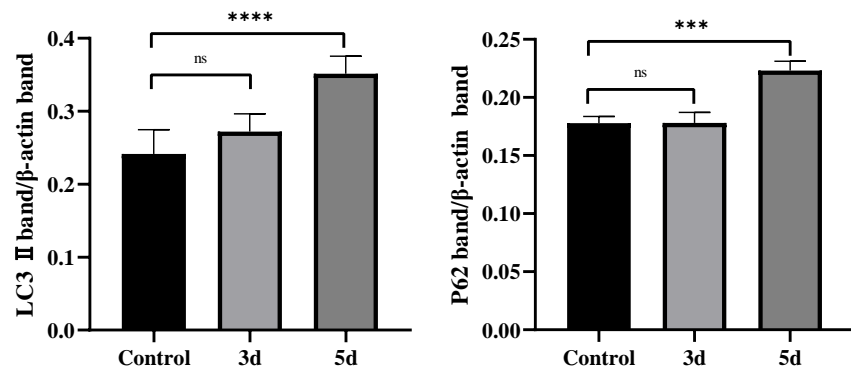

B

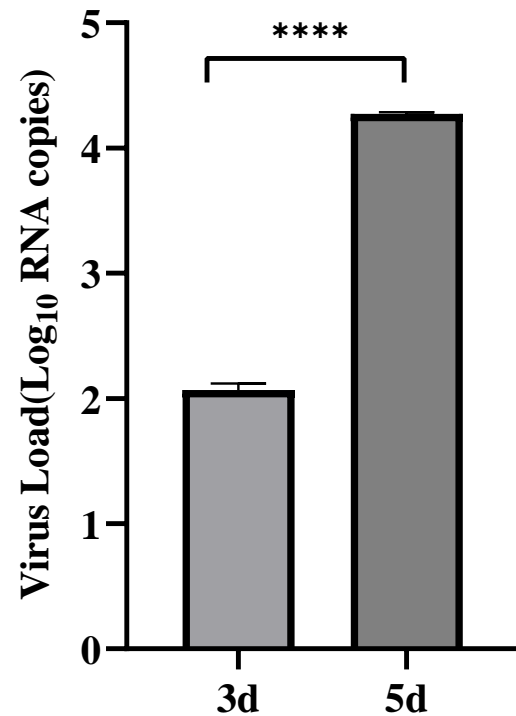

Supplement: Supplementary file 2 — Additional file 2: DTMUV AQ-19 infection induces incomplete autophagy in the goose brain. A Protein levels of LC3-II and P62 in the brain tissues of geese infected with DTMUV for 3 dpi and 5 dpi were determined by Western blotting, and band intensities were analysed. B qRT-PCR analysis of DTMUV copies in the brains of geese infected with AQ-19. The results are presented as the means ± SDs of three experiments. Differences were analysed using one-way ANOVA (A) and Student’s t test (B). ns: not significant; ***P < 0.001; ****P < 0.0001. [file 13567_2023_1235_MOESM2_ESM.pdf]
